# Enhanced antibiotic multi-resistance in nasal and faecal bacteria after agricultural use of streptomycin

**DOI:** 10.1111/1462-2920.12028

**Published:** 2012-11-15

**Authors:** Alexandre Scherer, Hans-Rudolf Vogt, Edy M Vilei, Joachim Frey, Vincent Perreten

**Affiliations:** 1 Institute of Veterinary Bacteriology, Vetsuisse Faculty, University of BernBern, Switzerland; 2 Institute of Virology, Vetsuisse Faculty, University of BernBern, Switzerland

## Abstract

Streptomycin is used in arboriculture to control fire blight. Using sheep as a model, multidrug-resistant bacteria in mammals were found to be selected after the intentional release of streptomycin into the environment. *Escherichia coli* and *Staphylococcus* spp. were isolated from the faeces and nasal cavities, respectively, of sheep grazing on a field sprayed with streptomycin at concentrations used in orchards (test group) and on a field without streptomycin (control group). Before the application of streptomycin, the percentage of streptomycin-resistant *E. coli* isolates in faeces was 15.8% in the control group and 14.7% in the test group. After the application of streptomycin, the overall number of streptomycin-resistant *E. coli* isolates was significantly higher in the test group (39.9%) than in the control group (22.3%). Streptomycin-resistant *Staphylococcus* isolates were only detected after the application of streptomycin. Streptomycin resistance was frequently associated with resistance to sulfamethoxazole, ampicillin, tetracycline and chloramphenicol and less frequently to cefotaxime in *E. coli*, and to tetracycline, fusidic acid and tiamulin in *Staphylococcus* spp. This study shows that the application of low concentrations of streptomycin on grass, as occurs during the spraying of orchards, selects for multidrug-resistant nasal and enteric bacterial flora, including extended-spectrum beta-lactamase-producing *E. coli*.

## Introduction

The number of multi-resistant bacterial strains has been increasing at an alarming rate and represents a major threat to modern medicine (Davies and Davies, 2010; D'Costa *et al*., 2011). The sharp increase in antibiotic resistance is caused by the extensive use of antibiotics in human and animal medicine and in agriculture (Neu, 1992; McManus *et al*., 2002; English and Gaur, 2010; Gootz, 2010; Wright, 2010). Of particular concern are community-acquired bacterial infections, such as those caused by methicillin-resistant *Staphylococcus aureus* and third-generation cephalosporin-resistant *Enterobacteriaceae* (Levy and Marshall, 2004; Bush, 2010; Cataldo *et al*., 2010; French, 2010; Fitzgerald, 2012; Pitout, 2012). Preventive measures to reduce the generation of antibiotic-resistant bacteria have been implemented and include banning the use of all antibiotics as growth promoters in animal breeding in Switzerland in 1999 and in the European Union in 2006 (Perreten, 2003; Cogliani *et al*., 2011). However, the impact of the agricultural use of streptomycin for plant protection on the selection of antibiotic-resistant bacteria in mammals has not been fully considered.

Fire blight, which is caused by *Erwinia amylovora*, a bacterium belonging to the *Enterobacteriaceae* family, is a serious bacterial disease affecting apple and pear trees and other rosaceous plants (Oh and Beer, 2005). To control this bacterial disease in orchards, producers are permitted to spray antibiotics directly on open blossoms after obtaining a special authorization from the agriculture authorities and following official application protocols (McManus *et al*., 2002; Mayerhofer *et al*., 2009). Since this practice began, streptomycin resistance in *E. amylovora* has been reported (Neu, 1992; McManus *et al*., 2002), arising from either a point mutation in the *rpsL* gene encoding the ribosomal S12 protein (McGhee *et al*., 2011) or the acquisition of mobile elements such as transposon Tn*5393*, which contains the streptomycin resistance genes *strA* [*aph(6)-Ia*] and *strB* [*aph(6)-Id*] (Chiou and Jones, [Bibr b9]; McGhee and Jones, 2000; McGhee *et al*., 2011). This element has also been found in *Salmonella enterica*, indicating the transfer of this element between *Enterobacteriaceae* (Pezzella *et al*., 2004). Sub-inhibitory concentrations of antibiotics, including streptomycin, have been shown to select efficiently for resistant bacteria (Cantón and Morosini, 2011; Gullberg *et al*., 2011). To determine the impact of streptomycin use in orchards on the nasal and intestinal flora of grazing animals, we studied the evolution of antibiotic resistance in *Escherichia coli* and *Staphylococcus* spp. from sheep that have grazed on grass sprayed with streptomycin at concentrations used against fire blight in orchards.

## Results

A total of 455 *E. coli* isolates (213 from the test group and 242 from the control group) from the faeces of sheep and 184 *Staphylococcus* spp. (86 from the test group and 98 from the control group) from nasal cavities were isolated during the 3 month period of monitoring. *Staphylococcus* species included 112 *Staphylococcus xylosus*, 15 *Staphylococcus vitulinus*, 14 *Staphylococcus sciuri*, 12 *Staphylococcus cohnii*, 9 *Staphylococcus simulans*, 8 *Staphylococcus* spp., 4 *Staphylococcus equorum*, 3 *Staphylococcus lentus*, 2 *Staphylococcus auricularis*, 2 *Staphylococcus hominis* and 1 *Staphylococcus epidermidis*. *Escherichia coli* were detected in all faecal samples, and staphylococci were detected in 72 of 80 nasal samples. One sheep from each group was excluded at day 43 due to the parasite disease hemonchosis.

**Fig. 1:**
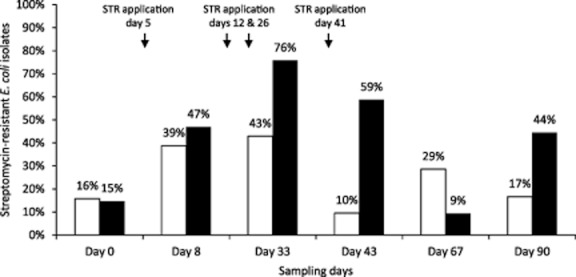
Percentage of streptomycin-resistant *E. coli* isolated each sampling day from sheep grazing on the pasture sprayed with streptomycin (black columns) and from control sheep grazing on the untreated pasture (white). STR, streptomycin.

Prior to the application of streptomycin to the field, 15.8% of the *E. coli* isolates from the faeces of the control group and 14.7% from the test group were resistant to streptomycin (Fig. 1). After the first application of streptomycin, the percentage of streptomycin-resistant *E. coli* isolates increased in both groups. The percentage of streptomycin-resistant *E. coli* was significantly higher in the study group than in the control group after the third and fourth application of streptomycin (Fig. 1). The percentage of streptomycin-resistant *E. coli* isolates unexpectedly increased to 28.6% in the control group 26 days after the last application. However, this increase was not statistically significant compared with the baseline percentage of streptomycin-resistant isolates that were present in the faeces of sheep before the first application of streptomycin (*P*-value 0.1922). The percentage of streptomycin-resistant *E. coli* was 44.4% in the test group and 16.7% in the control group 49 days after the last application (Fig. 1). The total number of streptomycin-resistant isolates after the application of streptomycin was significantly higher in the test group (39.9%, *n* = 85 of 213) than in the negative control group (22.3%, *n* = 54 of 242) (*P* = 0.0001).

**Fig. 2:**
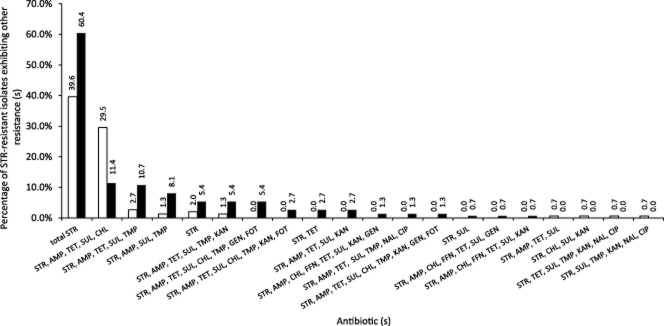
Distribution of multidrug resistance patterns in streptomycin-resistant *E. coli* isolated from the faeces of sheep during the 3 month monitoring period. Black columns: test group; white columns: negative control group. AMP, ampicillin; CIP, ciprofloxacin; CHL, chloramphenicol; FFN, florfenicol; FOT, cefotaxime; GEN, gentamicin; KAN, kanamycin; NAL, nalidixic acid; STR, streptomycin; SUL, sulfamethoxazole; TET, tetracycline; TMP, trimethoprim.

Resistance to streptomycin was linked to additional resistance in 92.6% of the streptomycin-resistant isolates, which were resistant to 1–9 antibiotics simultaneously (Fig. 2). Resistance to streptomycin was most often linked to resistance to sulfamethoxazole, ampicillin, tetracycline and chloramphenicol. Other frequent combinations of resistance to streptomycin and several antibiotics are shown in Fig. 2. Notably, streptomycin-resistant *E. coli* isolates from two sheep from the test group exhibited a multi-resistance profile that included resistance to the third-generation cephalosporin (3GC) cefotaxime (Fig. 2). Resistance to 3GC was attributed to the presence of the extended-spectrum beta-lactamase CTX-M-1. CTX-M-1 was only detected in one streptomycin-susceptible isolate from one sheep in the control group. Multidrug resistance associated with streptomycin was more frequent in *E. coli* isolates from sheep in the test group (40.4%, *n* = 86 of 213) than from sheep in the control group (23.9%, *n* = 58 of 242) (*P* = 0.0002), except for one multidrug resistance profile (streptomycin–sulfamethoxazole–ampicillin–tetracycline–chloramphenicol), which was more frequent in isolates from the control group (Fig. 2).

To analyse the genetic nature of streptomycin resistance in single- and multi-resistant strains, we randomly selected one *E. coli* isolate for each resistance profile and screened for the presence of streptomycin resistance genes by real-time PCR using primers (Table 1) designed from sequence alignments of the *aadA*, *str*, *strA*, *strB* and *ant(6)-Ia* genes available in GenBank (Fig. S1, Table S1). The streptomycin resistance genes *strA* and *strB* and genes belonging to either *aadA4* or *aadA5* or to *aadA1*, *aadA2*, *aadA3*, *aadA8*, *aadA12*, *aadA13*, *aadA15*, *aadA17*, *aadA21*, *aadA22*, *aadA23* or *aadA24* (*aadA*mix) were the most frequently detected genes among the isolates tested (Table 2). A class 1 integron (*intI1* gene) was also frequently detected, suggesting the presence of mobile elements in these *E. coli* isolates (Mazel, 2006). However, the association between class 1 integron and streptomycin resistance genes on the same genetic elements remains to be determined. In several isolates that exhibited resistance to streptomycin, no streptomycin resistance genes were detected (Table 2). These isolates also did not contain any mutations of the lysine residues K42 and K87 in RpsL, suggesting the presence of other streptomycin resistance mechanisms.

**Table 1:** Real-time PCR primers and probes used for the detection of antibiotic resistance genes in *E. coli* strains

Gene	Primer/probe name	Sequence (5′→3′)	Final concentration
*aadA1*	aadAmix_F	TTGCTIGCCGTICATTT	800 nM
*aadA2*			
*aadA3*			
*aadA8*			
*aadA12*	aadAmix_R	TCAATATCICTGTITGGCTTCAG	800 nM
*aadA13*			
*aadA15*			
*aadA17*			
*aadA21*	aadAmix_P	Cy5-TACGGCTCCGCAGTGGATGG-BHQ-2	100 nM
*aadA4*	aadA4_5_F	CATTGCTCCTAAGGACGTTGCT	20 nM
*aadA5*	aadA4_5_R	GATGCTCGGCAGGCAAAC	20 nM
	aadA4_5_P	VIC-CCGCATGGGTATCG-MGB	100 nM
*aadA6*	aadA6_11_16_F	CCAGACGGGAACTGCAATTC	100 nM
*aadA11*	aadA6_11_16_R	AGCCAGATCAACATCGGTTGT	100 nM
*aadA16*	aadA6_11_16_P	VIC-AAGGACATTCTTGCGGGC-MGB	100 nM
*aadA7*	aadA7_F	CCGCGCCTTGGAAGTG	20 nM
	aadA7_R	GCCGGATAACGCCAAGGT	20 nM
	aadA7_P	FAM-CCATCGTCGTGCACAGTGACATCG-TAMRA	200 nM
*aadA9*	aadA9_F	GAGGAATCGTCCCCAAGGA	700 nM
	aadA9_R	TCAGCTGGCAAGCGCTCTA	700 nM
	aadA9_P	Cy5-TGGCCGCCGAATGGGTT-BHQ-2	300 nM
*aadA10*	aadA10_F	CGCACGGCTCGATGAGA	80 nM
	aadA10_R	AAAACGGAAACCCCCAAGAG	80 nM
	aadA10_P	FAM-TGCGGCAAGCTCTGTTCGTCGA-TAMRA	250 nM
*aadA14*	aadA14_F	GTCCGATTTGTTGGCGGTAT	20 nM
	aadA14_R	CAAGTGCACGGCGTTTTG	20 nM
	aadA14_P	Cy5-CGCTTTCCCTGGCACCGA-BHQ-2	250 nM
*ant(6)-Ia*	ant6_F	GACATAGTTCCGACTGATATAGATTATCATG	300 nM
	ant6_R	GTGTTACATTCCAAAATTCATTGCA	300 nM
	ant6_P	VIC-AAGAAAGCCAAGCGC-MGB	150 nM
*intI1*	intI1_F	GCTTGTTCTACGGCACGTTTG	100 nM
	intI1_R	TGCGTCGCCATCACATGT	100 nM
	intI1_P	Cy5-AGGCGCGCTGAAAGGTCTGGTCA-BHQ-2	300 nM
*str*	str_F	GGTTAAAAAAACCAACAGAACGAGAA	80 nM
	str_R	CTAAAAACACCCTTTGCTACATACGT	80 nM
	str_P	VIC-ATGAGTTTTGGAGTGTCTC-MGB	250 nM
*strA*	strA_F	TGGCACTCATGATTGCTAACG	80 nM
	strA_R	GGCGCGCTCTGCTTCA	80 nM
	strA_P	FAM-CGAAGAGAACTGGGCAGCGCC-TAMRA	250 nM
*strB*	strB_F	ACAGAGACGACCTTTGTCTCGAT	100 nM
	strB_R	TCCAGCGCACGAGAGAATG	100 nM
	strB_P	Cy5-CTAGACGCATTGCACAGATGGCG-BHQ-2	250 nM
23S rRNA	23S TM-L	GITACIICGGGGATAACAGGC	900 nM
	23S TM-R	GCGAACAGCCIIACCCTTG	900 nM
	23S TM-S	FAM-TGGCACCTCGATGTCGGCTCITC-TAMRA	200 nM

The following combinations of primers and probes were used for multiplex PCRs: multiplex 1: *str*, *strA*, *strB*; multiplex 2: 23S rRNA (control for bacterial DNA), *intI1*, *aadA6_11_16*; multiplex 3: *aadA7*, *aadA9*, *aadA4_*5; multiplex 4: *aadA10*, *aadA14*, *ant(6)-Ia*; multiplex 5: *aadAmix*, IPC [exogenous internal positive control (VIC probe); Applied Biosystems, Forster City, CA, USA].

**Table 2:** Detection of streptomycin resistance genes and class 1 integron in *E. coli* isolates representative of each resistance profile

Antibiotic resistance profile	Genes detected
STR	–
STR, SUL	*strA*, *strB*
STR, AMP, TET	*strA*, *strB*
STR, AMP, TMP, SUL	–
STR, AMP, CHL, TET, SUL	*intI1*, *aadAmix*
STR, AMP, CHL, TET, KAN, SUL	*intI1*, *aadAmix*
STR, AMP, CHL, GEN, TET, SUL, KAN	*intI1*, *strA*, *strB*, *aadAmix*
STR, AMP, CHL, TMP, TET, SUL, KAN, FOT	*intI1*, *strA*, *strB*, *aadA5*

STR, streptomycin; SUL, sulfamethoxazole; AMP, ampicillin; TET, tetracycline; TMP, trimethoprim; CHL, chloramphenicol; GEN, gentamicin; KAN, kanamycin; FOT, cefotaxime.

**Fig. 3:**
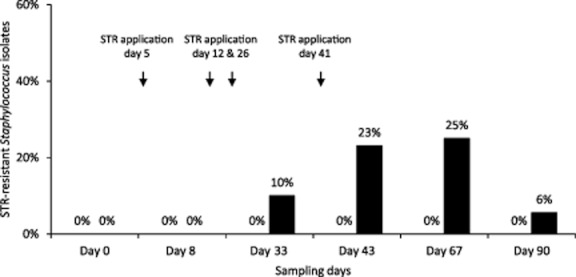
Percentage of streptomycin-resistant *Staphylococcus* spp. isolated each sampling day from sheep grazing on the pasture sprayed with streptomycin (black columns) and from control sheep grazing on the untreated pasture (white). STR, streptomycin.

Streptomycin-resistant *Staphylococcus* isolates from the nasal cavities were isolated only from sheep in the test group and only after application of streptomycin to the pasture (Fig. 3). In the test group, streptomycin-resistant *Staphylococcus* spp. emerged after the third application on day 33 for a total of 9.3% streptomycin-resistant isolates (*n* = 8 of 86) after spraying compared with none in the control group, representing a significant increase in streptomycin-resistant *Staphylococcus* spp. after treatment with streptomycin (*P* = 0.001). Among the eight streptomycin-resistant *Staphylococcus* isolates, two also exhibited additional resistance to tetracycline, fusidic acid and tiamulin, two to tetracycline and fusidic acid, two to fusidic acid and tiamulin, and two to fusidic acid.

## Discussion

*Escherichia coli* was revealed to be a good faecal indicator bacterium to monitor antibiotic resistance because it was present in all of the animals screened, whereas *Staphylococcus* spp. could not always be isolated from nasal samples. However, at least 12 of 14 sheep were positive for *Staphylococcus* spp. at each stage of sampling, allowing us to follow the development of resistance over the 3 months of monitoring. Standard medical methods recommended for bacterial infection and antibiotic resistance analysis have been prioritized over the cultivation-independent methods for detection of antimicrobial resistance genes in order to attribute the resistance profile to a specific bacterium and not to a population, and hence also identify multidrug resistance in the particular isolates.

Fluctuations in the percentage of streptomycin-resistant *E. coli* isolates were observed in both the test and control groups during the weeks following the last application of streptomycin to the field but in different proportions, with a much higher and significant increase in the test group than in the controls. Sheep from both the test and control groups may have acquired streptomycin-resistant bacteria already present in the soil as demonstrated by several studies (Thiele-Bruhn and Beck, 2005; Binh *et al*., 2007; 2009; Ding and He, 2010; Heuer *et al*., 2010; Byrne-Bailey *et al*., 2011). For instance, manure plays an important role in the release of antibiotics and antibiotic-resistant bacteria into the environment. These studies also demonstrated that sub-inhibitory concentrations of antibiotics in the soil as may occur after the application of manure select for specific antibiotic-resistant flora. Similarly, selection and enrichment of streptomycin-resistant bacteria most obviously occurred in the test group through the application of streptomycin, whereas selection in the control group may have occurred by minor spillover of streptomycin from the treated pasture by wind, insects, birds or other environmental factors because the two groups of sheep only were separated by a double fence with an interspace of 2 m such that conditions were as similar as possible for both groups. Although the fields used for the present study have not been fertilized with manure during the preceding 5 years, horses with a history of hospitalization and antibiotic treatment had regularly grazed in this area prior to the experiment. These animals may be one of the sources of residual antibiotic-resistant bacteria in the pastures used for our study (Schnellmann *et al*., 2006). Nevertheless, the number of streptomycin-resistant *E. coli* and *Staphylococcus* isolates was clearly higher in the test group, which is probably attributable to the application of streptomycin. This conclusion is also supported by the fact that streptomycin-resistant *Staphylococcus* spp. isolates were only isolated from sheep from the test group (Fig. 3) and appeared after the third application of streptomycin, whereas the number of streptomycin-resistant *E. coli* in the faeces of sheep increased after the first application. This result is probably due to an enrichment of the endogenous streptomycin-resistant *E. coli* already present in the gut, as described in other studies of the effects of antibiotics on the bacterial intestinal flora (Corpet, 1987; Khachatourians, 1998). The selection of streptomycin-resistant *E. coli* subpopulations, rather than *de novo* resistance, is also supported by the fact that the streptomycin-resistant flora increased rapidly, particularly in the test group. Additionally, the detection of streptomycin resistance genes known to be frequently located on transferrable genetic elements (Fluit and Schmitz, 1999) suggests that resistance may have the potential for dissemination to other bacteria. Indeed, low concentrations of antibiotics have been shown to efficiently select for gene transfer and antibiotic-resistant bacteria (Andersson and Hughes, 2011; Cantón and Morosini, 2011; Gullberg *et al*., 2011; Wu *et al*., 2011; Hughes and Andersson, 2012).

Our results show that the application of streptomycin to grass selects for multidrug-resistant flora in sheep, including resistance to antibiotics of major importance in human medicine, such as the cephalosporins. To the best of our knowledge, this is the first study that shows the effect of the agricultural use of streptomycin on animals grazing on fields contaminated with streptomycin. We demonstrate that the use of streptomycin in agriculture selects for multidrug-resistant bacteria in animals grazing on fields containing streptomycin at concentrations used to fight *E. amylovora* in orchards. To limit such a selection, authorities from countries like Switzerland and Austria require to prevent grazing of animals and forbid any hay harvest in orchards treated with antibiotics. However, it is anticipated that the use of streptomycin may also have effects similar to those observed in sheep on people working in streptomycin-treated orchards or living in their vicinity.

## Experimental procedures

### Study sites and sample collection

Four ewes (*Ovis aries*) and 10 3-month-old lambs (five female and five male), all belonging to the same breed (Swiss White Alpine Sheep), and which had never undergone antibiotic treatment, were placed together for 3 days and were separated into two flocks: a test group and a negative control group. Each flock containing two ewes and five lambs (arbitrarily selected) were placed into one of two distinct pastures of the Vetsuisse Faculty of Bern of approximately 2000 m^2^ each and separated by two fences situated 2 m apart. Due to the restricted area, the animals received extra feed and were treated twice with Dectomax (Pfizer, New York, USA) to prevent parasites.

The application of streptomycin followed the guidelines concerning the use of a phytosanitary product for the treatment of fire blight in orchards (Candolfi *et al*., 1998). Streptomycin was applied directly to the grass of the pasture of the test group with a 10 l spraying machine in four applications at day 5, day 12, day 26 and day 41. For each application, 4.6 g of streptomycin (Merck KGaA, Darmstadt, Germany) was sprayed on two adjacent surfaces of 3 m × 60 m and 2 m × 60 m at a concentration of 17.5 mg m^−2^ and 12.0 mg m^−2^, respectively, to mimic the gradient of streptomycin contamination resulting from the treatment of orchards. After the application, the sheep were confined to the sprayed area for 12 h and were released into the whole pasture. Anal and nasal swabs were taken from each sheep of both flocks, first from the negative control group and then from the test group at day 0, day 8 (3 days after the first streptomycin application), day 33 (6 days after the third application), day 43 (2 days after the fourth application), day 67 and day 90. No samples were taken after the second application at day 12 because of a period of heavy rain that followed the application.

### Bacterial isolation, identification and antibiotic susceptibility analysis

For the isolation of *E. coli*, anal swab samples were spread onto MacConkey agar (Oxoid, Basingstoke, UK) and incubated at 37°C for 24 h under aerobic conditions. At least four pink colonies from each sample were subcultured onto tryptone soy agar plates containing 5% sheep blood (TSA-SB) (Becton, Dickinson and Company, Franklin Lakes, NJ, USA) and incubated at 37°C for 24 h under aerobic conditions. Indol-positive and oxidase-negative isolates were confirmed as *E. coli* using MALDI-TOF-MS (Microflex LT; Bruker Daltonik, Bremen, Germany). *Staphylococcus* spp. were isolated from nasal swabs placed directly into tubes containing Mueller-Hinton broth (BBL™; Becton, Dickinson and Company, Franklin Lakes, NJ, USA) supplemented with 6.5% NaCl and incubated at 37°C for 24 h under agitation. Next, 10 μl of the culture was spread onto TSA-SB agar plates and onto *S. aureus* ID CHROMagar (SAID) (bioMérieux, Marcy l'Etoile, France) and incubated at 37°C for 24 h. One to three isolates exhibiting different morphologies and colours on SAID agar were Gram-stained and tested for catalase activity. Gram-positive cocci with catalase activity were identified using MALDI-TOF-MS and by sequence analysis of the *hsp60* gene (Goh *et al*., 1996). Minimal inhibitory concentrations (MIC) of antibiotics were determined in Mueller-Hinton broth using Sensititre susceptibility plates EUMVS2 for *E. coli* and EUST for staphylococci (Trek Diagnostics Systems, East Grinstead, UK; MCS Diagnostics BV, Swalmen, the Netherlands) according to CLSI guidelines (Clinical and Laboratory Standards Institute, 2009). Because no official resistance breakpoints exist for streptomycin, they were derived from epidemiological cut-off values and established as > 8 μg ml^−1^, as recently proposed for *E. coli* (Garcia-Migura *et al*., 2012) and at > 16 μg ml^−1^ for *Staphylococcus* spp. according to the European Committee on Antimicrobial Susceptibility Testing (http://www.eucast.org). Resistance breakpoints for the other antibiotics tested were those recommended by the CLSI (Clinical and Laboratory Standards Institute, 2012). Additional disc diffusion tests using cefoxitin (30 μg) (Oxoid, Basingstoke, UK) were performed on Mueller-Hinton agar (Difco™; Becton, Dickinson and Company) to screen for methicillin resistance in *Staphylococcus* species.

### Genetic analyses

Genes known to confer resistance to streptomycin as well as the class 1 integron were used as target for the TaqMan real-time PCR. The 23S rRNA was used as control for detection of bacterial DNA (Table 1). For each gene, a few disparities have been established and a consensus of homologue sequences has been designed (Table S1, Fig. S1). The different genes were aligned with sequences from the GenBank (Table S1, Fig. S1) using the Basic Local Alignment Search Tool (BLAST) nucleotide Program blastn (http://blast.ncbi.nlm.nih.gov). Similar sequences were aligned using the ClustalW Program (http://www.ch.embnet.org) and MultAlin Program (http://multalin.toulouse.inra.fr/multalin/). The oligonucleotide primers and fluorogenic probe for each consensus of sequence were designed using the Primer express 3.0 software (Applied Biosystems, Foster City, CA, USA). The potential amplicons with the lowest penalty (determined with the primer express software) were chosen and entered in the program blastn to confirm the specificity. Only potential amplicons with no similarities to other targets were selected. TaqMan real-time PCR was performed on a 7500 Fast Real-Time PCR System (Applied Biosystems). Each reaction contained 1 × TaqMan Universal PCR Master Mix No AmpErase UNG (Applied Biosystems), each TaqMan primer and TaqMan probe at different concentrations (Table 1), and 2.5 μl of template DNA (at a concentration of 4 to 40 ng μl^−1^) to a total volume of 25 μl. The real-time PCR program consisted of a first hold step at 50°C for 2 min and a second hold step at 95°C for 10 min followed by 40 cycles of 95°C for 15 s and 60°C for 1 min. The reactions were performed in multiplex combinations consisting of four triplexes and one duplex (Table 1) and validated with positive control reference strains. Presence of mutations in the *rpsL* gene was determined by sequence analysis of PCR products amplified as described previously (Toivonen *et al*., 1999). Extended-spectrum beta-lactamase genes were identified by sequence analysis of PCR products (Pitout *et al*., 2004).

### Statistic methods

NCSS 2007 (Kaysville, UT, USA) was used to conduct a Fisher's exact test (two-tailed) with the level of significance set at a *P*-value < 0.05 and to apply the exact binomial approach for the calculation of confidence intervals (CI).

## Supporting information

**Fig. S1.** Homology tree of *aadA* genes with subgroups used as common targets for real-time PCR. Subgroups were generated with sequences with > 85% similarities. The tree was generated using UPGMA [Pairwise (OG: 100%, UG: 0%) Gapcost: 0%] using Bionumerics 5.10 (Applied Maths, Sint-Martens-Latem, Belgium). The number designations of the different *aadA* genes correspond to their respective entries in GenBank. The letter designations of the different *aadA* genes were proposed by the present study.

**Table S1.** Streptomycin resistance and integrase genes used to design primers and probes for the TaqMan real-time PCR.
