# Supplementary material for: Enhanced antibiotic multi-resistance in nasal and faecal bacteria after agricultural use of streptomycin
Source: Environ Microbiol. 2012 Nov 15;15(1):297–304. doi: 10.1111/1462-2920.12028 (PMC3558797; doi:10.1111/1462-2920.12028)
Supplement: Supplementary file 1 [file emi0015-0297-SD1.pptx]

## Slide 1
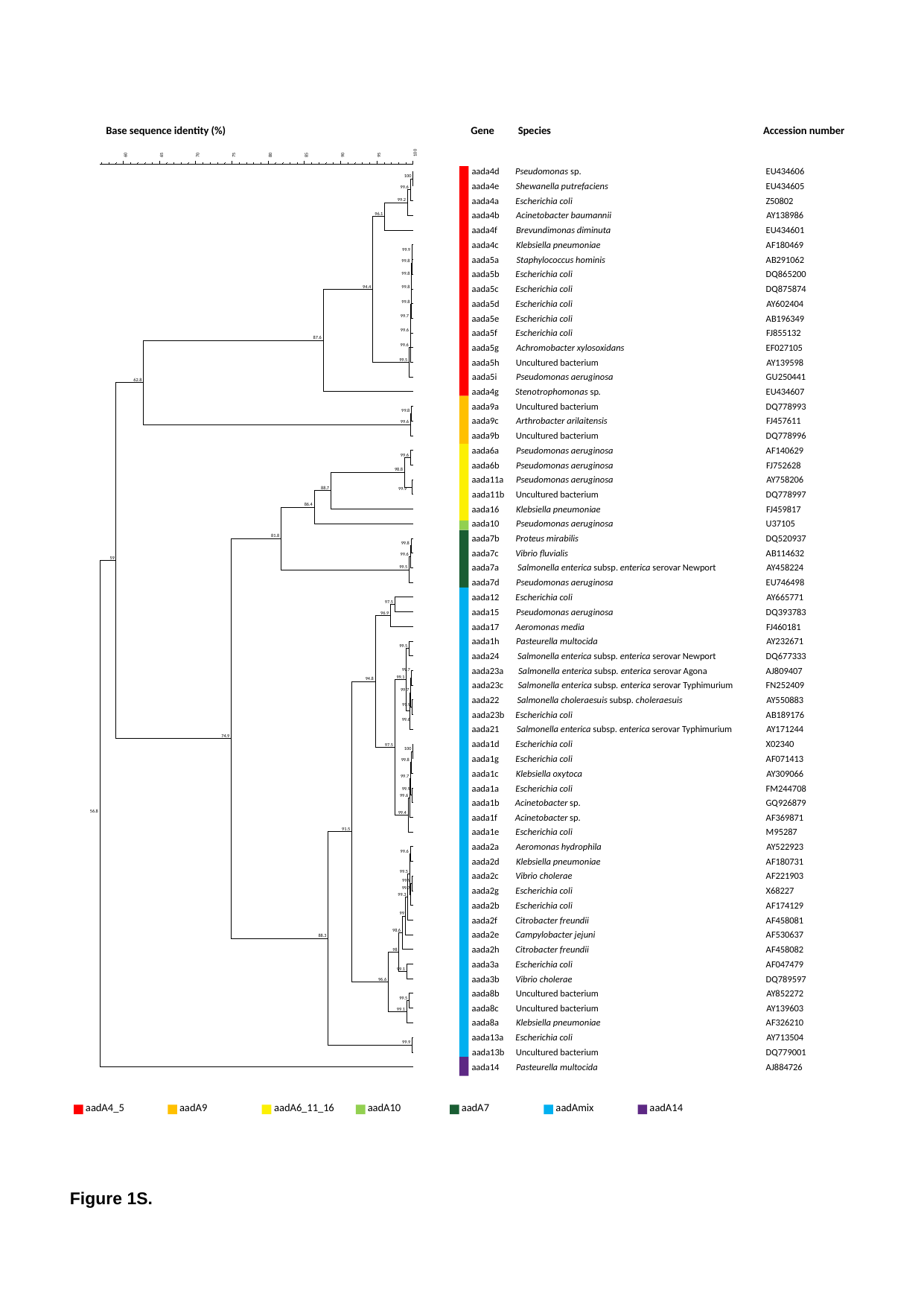

Base sequence identity (%)
Gene
Species
Accession number
100
60
65
70
75
80
85
90
95
100
99.6
99.2
96.1
99.9
99.8
99.8
94.4
99.8
99.8
99.7
99.6
87.6
99.6
99.5
62.8
99.8
99.6
99.6
98.8
88.7
99.9
86.4
81.8
99.8
99.6
59
99.5
97.5
96.9
99.5
99.7
99.1
94.8
99.7
99.9
99.6
74.9
97.5
100
99.8
99.7
99.9
99.6
56.8
99.4
91.5
99.6
99.5
99.9
99.7
99.3
99
98.6
88.3
98
99.1
96.6
99.5
99.1
99.9
aada4d
Pseudomonas sp.
EU434606
aada4e
Shewanella putrefaciens
EU434605
aada4a
Escherichia coli
Z50802
aada4b
Acinetobacter baumannii
AY138986
aada4f
Brevundimonas diminuta
EU434601
aada4c
Klebsiella pneumoniae
AF180469
aada5a
Staphylococcus hominis
AB291062
aada5b
Escherichia coli
DQ865200
aada5c
Escherichia coli
DQ875874
aada5d
Escherichia coli
AY602404
aada5e
Escherichia coli
AB196349
aada5f
Escherichia coli
FJ855132
aada5g
Achromobacter xylosoxidans
EF027105
aada5h
Uncultured bacterium
AY139598
aada5i
Pseudomonas aeruginosa
GU250441
aada4g
Stenotrophomonas sp.
EU434607
aada9a
Uncultured bacterium
DQ778993
aada9c
Arthrobacter arilaitensis
FJ457611
aada9b
Uncultured bacterium
DQ778996
aada6a
Pseudomonas aeruginosa
AF140629
aada6b
Pseudomonas aeruginosa
FJ752628
aada11a
Pseudomonas aeruginosa
AY758206
aada11b
Uncultured bacterium
DQ778997
aada16
Klebsiella pneumoniae
FJ459817
aada10
Pseudomonas aeruginosa
U37105
aada7b
Proteus mirabilis
DQ520937
aada7c
Vibrio fluvialis
AB114632
aada7a
Salmonella enterica subsp. enterica serovar Newport
AY458224
aada7d
Pseudomonas aeruginosa
EU746498
aada12
Escherichia coli
AY665771
aada15
Pseudomonas aeruginosa
DQ393783
aada17
Aeromonas media
FJ460181
aada1h
Pasteurella multocida
AY232671
aada24
Salmonella enterica subsp. enterica serovar Newport
DQ677333
aada23a
Salmonella enterica subsp. enterica serovar Agona
AJ809407
aada23c
Salmonella enterica subsp. enterica serovar Typhimurium
aada22
Salmonella choleraesuis subsp. choleraesuis
aada23b
Escherichia coli
aada21
Salmonella enterica subsp. enterica serovar Typhimurium
aada1d
Escherichia coli
aada1g
Escherichia coli
aada1c
Klebsiella oxytoca
Escherichia coli
aada1a
aada1b
Acinetobacter sp.
aada1f
Acinetobacter sp.
aada1e
Escherichia coli
aada2a
Aeromonas hydrophila
aada2d
Klebsiella pneumoniae
aada2c
Vibrio cholerae
Escherichia coli
aada2g
aada2b
Escherichia coli
aada2f
Citrobacter freundii
aada2e
Campylobacter jejuni
aada2h
Citrobacter freundii
aada3a
Escherichia coli
aada3b
Vibrio cholerae
aada8b
Uncultured bacterium
aada8c
Uncultured bacterium
aada8a
Klebsiella pneumoniae
aada13a
Escherichia coli
aada13b
Uncultured bacterium
aada14
Pasteurella multocida
FN252409
AY550883
AB189176
AY171244
X02340
AF071413
AY309066
FM244708
GQ926879
AF369871
M95287
AY522923
AF180731
AF221903
X68227
AF174129
AF458081
AF530637
AF458082
AF047479
DQ789597
AY852272
AY139603
AF326210
AY713504
DQ779001
AJ884726
aadA4_5
aadA9
aadA6_11_16
aadA10
aadA7
aadAmix
aadA14
Figure 1S.
