# Supplementary material for: Enhanced antibiotic multi-resistance in nasal and faecal bacteria after agricultural use of streptomycin
Source: Environ Microbiol. 2012 Nov 15;15(1):297–304. doi: 10.1111/1462-2920.12028 (PMC3558797; doi:10.1111/1462-2920.12028)
Supplement: Supplementary file 2 [file emi0015-0297-SD2.docx]

**Table 1S.** Streptomycin resistance and integrase genes used to design primers and probes for the TaqMan real-time PCR.

| Gene | Reference strain | Accession number |
| --- | --- | --- |
| *ant(6)-Ia* | *Enterococcus faecium* | AF516335 |
|  | *Enterococcus faecalis* | AB247327 |
|  | *Streptococcus oralis* | AY712687 |
|  | *Enterococcus faecalis* | AY626923 |
|  | *Enterococcus faecium* | FJ441296 |
|  | *Enterococcus faecium* | FJ441294 |
|  | *Enterococcus faecalis* | [FJ441295](http://www.ncbi.nlm.nih.gov/nucleotide/215490331?report=genbank&log$=nucltop&blast_rank=51&RID=HK7BTZVG011) |
| *intI1* | *Escherichia coli* | [FJ763641](http://www.ncbi.nlm.nih.gov/nucleotide/225730209?report=genbank&log$=nucltop&blast_rank=12&RID=HK85BJPX01N) |
|  | *Escherichia coli* | FJ196385 |
|  | *Xanthomonas oryzae* | FJ501976 |
|  | *Enterobacter cloacae* | [EU855788](http://www.ncbi.nlm.nih.gov/nucleotide/226426103?report=genbank&log$=nucltop&blast_rank=96&RID=HK85BJPX01N) |
|  | *Acinetobacter baumannii* | CT025832 |
|  | *Salmonella enterica* | EU219534 |
|  | *Klebsiella pneumonia* | AJ704863 |
|  | *Aeromonas salmonicida* | [CP000645](http://www.ncbi.nlm.nih.gov/nucleotide/142855988?report=genbank&log$=nucltop&blast_rank=118&RID=HK8B9T2P011) |
| *str* | *Staphylococcus aureus* | X06627 |
|  | *Lactococcus lactis* subsp. *lactis* | X92946 |
|  | *Staphylococcus intermedius* | AY604739 |
|  | *Enterococcus casseliflavus* | AY939911 |
|  | *Macrococcus caseolyticus* | AP009489 |
| *strA* | *Actinobacillus porcitonsillarum* | AM748706 |
|  | *Pasteurella multocida* | FJ197818 |
|  | *Haemophilus parasuis* | FJ670543 |
|  | Uncultured bacterium | FJ012880 |
|  | *Mannheimia varigena* | AJ319822 |
|  | *Edwardsellia tarda* | CP001136 |
| *strB* | *Escherichia coli* | M28829 |
|  | *Escherichia coli* | FJ968160 |
|  | *Escherichia coli* | AJ313522 |
|  | *Vibrio cholera* | AY958065 |
|  | *Acinetobacter baumannii* | CU459141 |
|  | *Vibrio cholerae* | FJ750808 |
